# Impact of Pretransplant Renal Replacement Therapy on Clinical Outcome After Isolated Heart Transplantation

**DOI:** 10.3389/ti.2022.10185

**Published:** 2022-03-21

**Authors:** Jeng-Wei Chen, Nai-Kuan Chou, Chih-Hsien Wang, Nai-Hsin Chi, Shu-Chien Huang, Hsi-Yu Yu, Yih-Sharng Chen, Ron-Bin Hsu

**Affiliations:** ^1^ Division of Cardiovascular Surgery, Department of Surgery, National Taiwan University Hospital, National Taiwan University College of Medicine, Taipei, Taiwan; ^2^ Graduate Institute of Clinical Medicine, College of Medicine, National Taiwan University, Taipei, Taiwan

**Keywords:** acute kidney injury, renal replacement therapy, heart transplant, long term survival, renal failure

## Abstract

End stage renal disease (ESRD) is a contraindication to isolated heart transplantation (HT). However, heart candidates with cardiogenic shock may experience acute kidney injury and require renal replacement therapy (RRT) and isolated HT as a life-saving operation. The outcomes, including survival and renal function, are rarely reported. We enrolled 569 patients undergoing isolated HT from 1989 to 2018. Among them, 66 patients required RRT before HT (34 transient and 32 persistent). The survival was worse in patients with RRT than those without (65.2% vs 84.7%; 27.3% vs 51.1% at 1- and 10-year, *p* < 0.001 and *p* = 0.012, respectively). Multivariate Cox analysis identified pre-transplant hyperbilirubinemia (Hazard ratio (HR) 2.534, 95% confidence interval (CI) 1.098–5.853, *p* = 0.029), post-transplant RRT (HR 5.551, 95%CI 1.280–24.068, *p* = 0.022) and post-transplant early bloodstream infection (HR 3.014, 95%CI 1.270–7.152, *p* = 0.012) as independent risk factors of 1-year mortality. The majority of operative survivors (98%) displayed renal recovery after HT. Although patients with persistent or transient RRT before HT had a similar long-term survival, patients with persistent RRT developed a high incidence (49.2%) of dialysis-dependent ESRD at 10 years. In transplant candidates with pretransplant RRT, hyperbilirubinemia should be carefully re-evaluated for the eligibility of HT whereas prevention and management of bloodstream infection after HT improve survival.

## Graphical Abstract

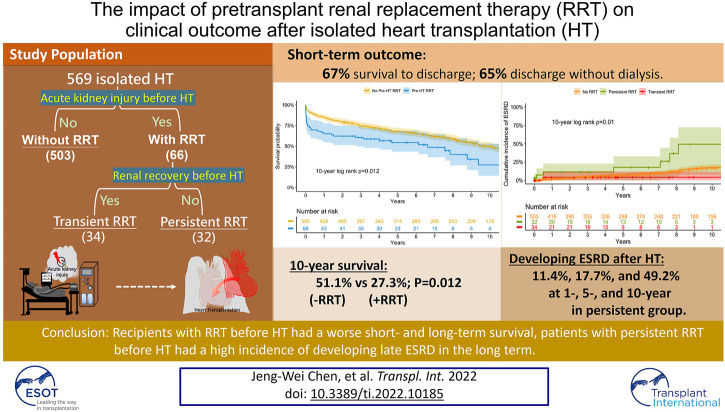

## Introduction

Heart transplant candidates with acute decompensated heart failure and complicating acute kidney injury (AKI) experience increased fluid overload and subsequent heart function deterioration before heart transplantation (HT) (1). With the advancement of mechanical circulatory support (MCS) and renal replacement therapy (RRT), more end‐stage heart failure patients could wait for HT with a bridge using MCS devices such as an extracorporeal membrane oxygenator (ECMO) or a ventricular assisted device (VAD) (2, 3). More than 30% of patients with MCS devices develop AKI and require RRT (4), The requirement of RRT before HT is one of the major risk factors of 1-year mortality after HT (5).

The renal outcome in patients with AKI requiring RRT before HT varies widely. Some patients have improved renal function quickly after achieving stable hemodynamics, while others require persistent RRT even after HT (6). Persistent RRT can increase the long-term mortality after HT (5).

Combined heart and kidney transplantation (HKT) has been recommended in heart candidates with comorbid renal dysfunctions (7, 8). However, candidates with AKI requiring RRT might receive isolated HT as a life-saving operation. As the clinical outcomes, including survival and renal function, in these patients, are rarely reported, we sought to investigate the impact of pretransplant RRT on clinical outcomes after isolated HT (5).

## Patients and Methods

**FIGURE 1:**
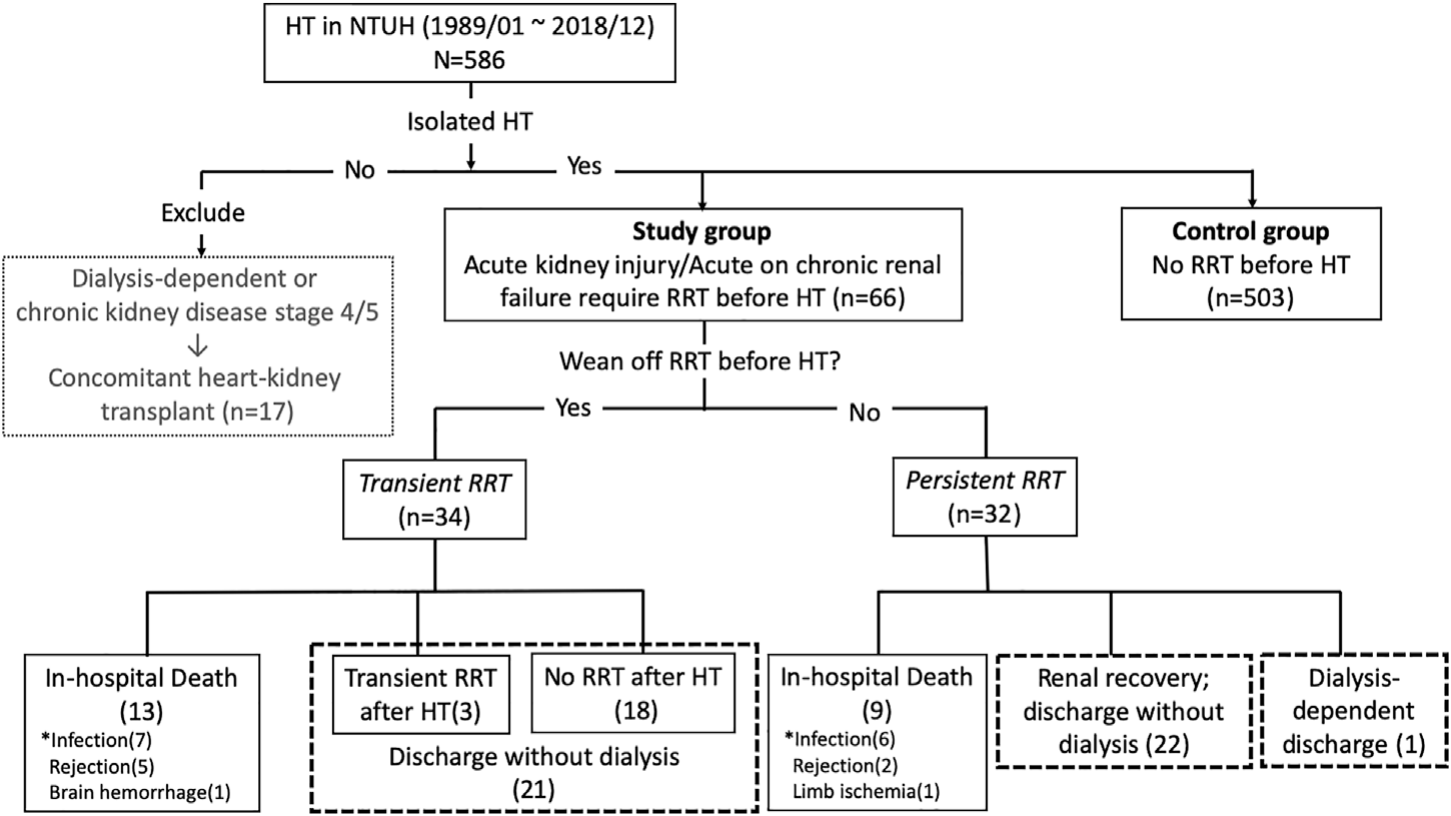
Details regarding the inclusion of study patients. RRT, renal replacement therapy; HT, heart transplantation.

The study protocol was approved by the Ethics Review Board of the National Taiwan University Hospital (202006034RINC). The requirement for informed consent was waived. This retrospective cohort study included patients who underwent HT in this hospital between January 1989 and December 2018 (Figure 1).

For heart candidates with stage 4 or 5 chronic kidney disease (CKD) or dialysis-dependent end stage renal disease (ESRD), transplant nephrologists were consulted for evaluation of combined HKT. Seventeen patients with combined HKT were excluded. However, heart candidates with AKI requiring RRT and who had the potential for renal recovery may have received isolated HT.

Indications for RRT included metabolic acidosis (pH < 7.2), electrolyte imbalance (potassium > 6.5 mmol/L), severe pulmonary edema, and fluid overload that was unresponsive to intravenous diuretics. All our patients received the less stressful continuous veno-venous hemofiltration as the first-choice modality of RRT until termination of RRT or HT. Two patients required intermittent hemodialysis for inadequate urine output after stopping continuous veno-venous hemofiltration with stable hemodynamics. A nephrologist was regularly consulted for renal function evaluation. Each patient’s urine output, fluid status, and biochemical data were evaluated daily to assess renal recovery and facilitate the eventual RRT discontinuation. Patients with RRT before HT were divided into two subgroups according to whether they could be weaned from RRT before HT. We defined transient RRT as weaned from RRT before HT and persistent RRT as requiring RRT until HT.

### Mechanical Support Before HT

Policies regarding MCS before HT have been reported previously (9). In patients suffering from profound cardiogenic shock and who were potential candidates for HT, ECMO was applied to identify any complication that may have arisen as a result of resuscitation that would be a contraindication for HT. The relative and absolute contraindications of HT were followed according to the International Society for Heart and Lung Transplantation (ISHLT) listing criteria (9). After 4–5 days, VAD implantation was considered as a bridge to HT for patients who could not be weaned from ECMO.

### Immunosuppression After HT

All patients received triple-drug immunosuppressive therapy according to previously reported protocols (10,11, 12). Briefly, rabbit anti-thymocyte globulin was administered post-transplantation for 3–5 days. Cyclosporine was administered orally within 5 days of transplantation or after renal function recovery. To reduce nephrotoxicity, the cyclosporine dose was decreased to maintain a serum trough level of 250–350 ng/ml during the first 3 months and 150–250 ng/ml at 1 year. Azathioprine was administered post-transplantation, and the dose was adjusted to maintain a leukocyte count of 4,000–6,000/mm^3^. Prednisone (0.5 mg/kg/day) was administered postoperatively and tapered to 0.2 mg/kg/day by 1 month. Since 2004, mycophenolate mofetil has been used instead of azathioprine for primary immunosuppression (12, 13). Everolimus has been used for primary immunosuppression since 2010.

### Data Collection

Pre-transplant data including recipient’s characteristics, complicated bloodstream infection (BSI), and dialysis duration were collected by chart review. The baseline renal function was assessed upon admission. The estimated glomerular filtration rate was calculated using the Cockcroft-Gault formula (14). Perioperative data included the donor’s age and sex, and allograft ischemic time. Post-transplant data included mortality, date, and cause of death, date of the first dialysis, early BSI within 30-days after HT, and major postoperative complications.

### Statistical Analysis

All statistical analyses were performed using R (version 4.1.0; R Foundation for Statistical Computing, Vienna, Austria). The continuous variables were expressed as the median and interquartile range, and categorical variables were described by frequency values. Comparison of patients with and without RRT before HT was performed using Fisher’s exact test to compare categorical variables when observed frequencies were <5 in more than 25% of cells and Mann-Whitney *U* test for continuous variables. Subgroup analyses were performed in patients with transient RRT and patients with persistent RRT until HT. Cox proportional regression was used to identify independent risk factors of 1-year mortality in patients requiring RRT before HT and included all potential predictors with a *p*-value < 0.1 in the univariate analysis.

The results of multivariable models are reported as the hazard ratio with corresponding 95% confidence intervals. The cumulative incidence of survival curves and ESRD were plotted using the Kaplan-Meier method. The survival rates between groups were compared using the log-rank test. Competing risk analysis was carried out with cumulative incidence of ESRD and death before ESRD by the abovementioned groups. *p* values < 0.05 were considered statistically significant.

## Results

### Patient Demographics

This study enrolled 569 patients receiving isolated HT. Sixty-six patients requiring RRT before HT were compared with 503 patients without RRT before HT. Patients with RRT were further divided into two subgroups: 34 transient RRT and 32 persistent RRT.

Table 1 shows the basic patient demographics. The most common etiology of heart failure in patients with RRT was dilated cardiomyopathy (33%) followed by ischemic cardiomyopathy (29%). Patients with RRT were older and had a greater body weight, a higher incidence of diabetes, and a worse baseline renal function. More patients with RRT had United Network for Organ Sharing (UNOS) status 1A (89%), MCS (82%), resternotomy surgery (77%), and previous cardiopulmonary resuscitation (44%). Patients with persistent RRT had a higher rate of diabetes and an even worse baseline renal function than patients with transient RRT.

**TABLE 1:** Characteristics and clinical outcomes of patients with and without renal replacement therapy (RRT) before heart transplantation (HT).

Variables median (IQR); n (%)	Without RRT *n* = 503	With RRT *n* = 66	*p*-value	Subgroup^a^		
				Transient *n* = 34	Persistent *n* = 32	*p*-value
Age, year	49 (34–58)	51 (40–58)	0.025	52 (39–58)	51 (40–58)	0.948
Woman	84 (17)	10 (15)	0.750	3 (9)	7 (22)	0.180
Body weight, kilograms	62 (54–70)	67 (58–75)	0.002	70 (56–76)	65 (59–75)	0.735
Blood type			0.622			0.238
A	158 (31)	23 (35)		11 (32)	12 (38)	
B	140 (28)	19 (29)		10 (29)	9 (28)	
O	158 (31)	21 (32)		13 (38)	8 (25)	
AB	47 (9)	3 (4)		0	3 (9)	
Comorbidities						
Smoker	112 (22)	16 (24)	0.742	9 (26)	7 (22)	0.777
Hyperlipidemia	131 (26)	20 (30)	0.473	7 (21)	13 (41)	0.109
Diabetes	113 (22)	24 (36)	0.013	8 (24)	16 (50)	0.040
Etiology			<0.001			0.597
Dilated cardiomyopathy	263 (52)	22 (33)		10 (29)	6 (19)	
Ischemic cardiomyopathy	129 (26)	19 (29)		7 (21)	11 (34)	
Acute myocarditis	11 (2)	7 (11)		4 (12)	3 (9)	
Acute myocardial infarction	25 (5)	13 (20)		7 (21)	6 (19)	
Congenital heart disease	18 (4)	2 (3)		1 (3)	1 (3)	
Retransplantation	14 (3)	3 (4)		2 (6)	1 (3)	
Rheumatic heart disease	25 (5)	0		0	0	
Others	18 (4)	0		0	0	
Pre-transplant						
UNOS status 1A	139 (28)	59 (89)	<0.001	31 (91)	28 (88)	0.628
Mechanical ventilation	76 (15)	55 (83)	<0.001	29 (85)	26 (81)	0.748
Intra-aortic balloon pump	69 (14)	34 (52)	<0.001	16 (47)	18 (56)	0.473
Mechanical circulatory support	107 (21)	54 (82)	<0.001	30 (88)	24 (75)	0.210
ECMO	36 (7)	19 (29)	<0.001	9 (26)	10 (31)	0.668
Non-durable VAD ± ECMO	52 (10)	33 (50)	<0.001	19 (56)	14 (44)	0.325
Durable VAD	19 (4)	2 (3)	0.964	2 (6)	0	
Previous cardiopulmonary resuscitation	76 (15)	29 (44)	<0.001	15 (44)	14 (44)	1
Baseline renal function						
Creatinine, mg/dl	1.1 (0.9–1.5)	1.8 (1.1–2.5)	<0.001	1.4 (1.0–2.3)	2.0 (1.5–2.7)	0.015
Blood urea nitrogen, mg/dl	24 (17–34)	34 (24–54)	<0.001	32 (20–43)	41 (27–66)	0.264
eGFR, ml/min/1.73m^2^	68 (51–86)	42 (29–67)	<0.001	59 (31–82)	35 (27–47)	0.002
BSI within 2 weeks before HT	6 (1)	8 (12)	<0.001	4 (12)	4 (13)	1
Blood T-bil before HT, mg/dl	1.3 (0.8–2.3)	1.5 (0.9–3.8)	<0.001	1.4 (0.9–3.9)	1.6 (1.0–3.9)	0.646
Length of RRT, days	0	17 (7–35)	<0.001	16 (6–35)	18 (10–35)	0.675
Length of in-hospital waiting, days	2 (0–29)	36 (18–64)	<0.001	51 (23–88)	27 (13–46)	0.020
Donor characteristics						
Age, year	32 (22–44)	39 (28–47)	0.008	37 (28–47)	43 (29–50)	0.495
Body weight, kg	63 (55–70)	65 (58–77)	0.507	69 (60–79)	63 (55–70)	0.083
Woman	141 (28)	20 (31)	0.873	6 (18)	14 (44)	0.033
Intra-operative						
Resternotomy	151 (30)	46 (70)	<0.001	25 (74)	21 (66)	0.485
Allograft ischemic time, min	144 (100–211)	168 (127–228)	0.020	167 (130–212)	177 (116–237)	0.221
Post-transplant						
RRT	71 (14)	43 (65)	<0.001	19 (56)	24 (75)	0.103
Early BSI in 30-day	47 (9)	13 (20)	0.01	7 (21)	6 (19)	0.851
1-year mortality	77 (15)	23 (33)	<0.001	13 (38)	10 (31)	0.552
Follow-up duration, year	7.2 (2.7–12.2)	3.2 (0.1–6.6)	<0.001			

BSI, bloodstream infection; eGFR, estimated glomerular filtration rate; ECMO, extracorporeal membrane oxygenator; T-bil: total bilirubin; UNOS, united network for organ sharing; VAD, ventricular assisted device.

a Patients with RRT, before HT, were divided into two subgroups according to whether they could be weaned from RRT, before HT, or not: transient RRT (weaned from RRT, before HT) and persistent RRT (requiring RRT, until HT).

### Short-Term Outcomes

Hospital survival and renal outcome after HT were shown in Figure 1. In patients with RRT, 43 (65%) patients survived discharge without RRT: 21 (62%) and 22 (69%) patients in transient and persistent RRT subgroups, respectively. In the transient RRT subgroup, 3 required a short-term RRT after HT. In the persistent RRT subgroup, 1 was dialysis-dependant after discharge. There was no difference in the rate of survival to discharge without RRT between transient and persistent RRT subgroups.

As shown in Table 1, patients with RRT had a higher 1-year mortality rate than patients without RRT (15% versus 33%, *p* < 0.001). The most common cause of post-transplant death in patients without RRT was primary graft failure (10 of 30, 33%) within the first month and infection (20 of 47, 43%) from 1 month to 1 year. However, in patients with RRT, the most common cause of death was infection both within the first month (8 of 15, 53%) and from 1 month to 1 year (5 of 8, 62.5%) after HT.

Thirteen patients with RRT (20%) had 16 episodes of early BSI after HT, and the source was the wound for 5 patients (31%), the catheter for 3 (19%), pneumonia for 3 (19%), urosepsis for 1 (6%) and primary BSI for 4 (25%).

Table 2 showed the cox proportional analysis for the risk factors of 1-year mortality. Both univariate and multivariate analysis identified pre-transplant hyperbilirubinemia (serum total-bilirubin > 3 mg/dl) (hazard ratio (HR): 2.534, 95% confidence interval (CI): 1.098–5.853, *p* = 0.029), post-transplant RRT (HR: 5.551, 95% CI: 1.280–24.068, *p* = 0.022) and post-transplant early BSI (HR: 3.014, 95% CI: 1.270–7.152, *p* = 0.012) as significant risk factors of 1-year mortality after HT.

**TABLE 2:** Cox regression for 1-year mortality in patients requiring renal replacement therapy (RRT) before heart transplantation (HT).

Variable	Univariate			Multivariate			
	HR	95% CI	*p*-value	HR	95% CI		*p*-value
Recipient							
Age	1.012	0.979–1.046	0.482				
Woman	1.735	0.643–4.68	0.277				
Body weight, kilograms	0.98	0.951–1.01	0.18				
Blood type							
O	1						
A	0.646	0.24–1.737	0.387				
B	0.817	0.304–2.196	0.689				
Smoker	1.438	0.591–3.498	0.424				
Hyperlipidemia	0.761	0.3–1.933	0.566				
Diabetes	0.946	0.401–2.233	0.9				
Previous cardiopulmonary resuscitation	1.806	0.792–4.12	0.16				
Resternotomy	1.787	0.663–4.816	0.251				
UNOS status 1A	0.629	0.187–2.119	0.455				
Mechanical ventilator	0.902	0.307–2.652	0.851				
Intra-aortic balloon pump	0.845	0.373–1.914	0.686				
Mechanical circulatory support							
non-use	1						
ECMO	1.767	0.554–5.638	0.336				
Non-durable VAD ± ECMO	0.618	0.186–2.053	0.432				
Durable VAD	1.872	0.209–16.787	0.575				
Baseline renal function							
Creatinine, mg/dl	1.113	0.803–1.543	0.519				
Blood urea nitrogen, mg/dl	1.008	0.993–1.023	0.278				
eGFR, ml/min/1.73 m^2^	0.987	0.97–1.004	0.122				
Diagnosis							
Dilated cardiomyopathy	1						
Acute myocarditis	0.719	0.153–3.387	0.677				
Acute myocardial infarction	0.826	0.249–2.743	0.755				
Ischemic cardiomyopathy	1.084	0.393–2.989	0.877				
Congenital heart disease	2.164	0.27–17.328	0.467				
Retransplantation	1.062	0.133–8.493	0.955				
Pretransplant							
Persistent RRT	1.286	0.564–2.933	0.550				
Length of RRT, days	0.99	0.974–1.005	0.202				
Length of ECMO support, days	0.958	0.892–1.029	0.243				
Length of hospital-stay, days	0.995	0.984–1.005	0.336				
Blood total-bilirubin ≥ 3 mg/dl	3.198	1.405–7.280	0.006	2.534	1.098–5.853		0.029
BSI within 2 weeks	1.774	0.603–5.224	0.298				
Donor							
Age	1.003	0.971–1.036	0.869				
Woman	0.768	0.303–1.948	0.578				
Body weight, kilograms	0.995	0.964–1.027	0.755				
Allograft ischemic time, minutes	1.002	0.996–1.007	0.562				
Posttransplant							
RRT	7.260	1.699–31.015	0.007	5.551		1.280–24.068	0.022
Early BSI in 30-day	4.642	2.001–10.769	<0.001	3.014	1.270–7.152		0.012

BSI, bloodstream infection; CI, confidence interval; eGFR, estimated glomerular filtration rate; ECMO, extracorporeal membrane oxygenator; HR, hazard ratio; UNOS, united network for organ sharing; VAD, ventricular assisted device.

Patients with RRT had a higher incidence of receiving pre-transplant ECMO support than patients without RRT (74% versus 16%, *p* < 0.001). These incidences were not significantly different between the transient and persistent RRT subgroups (82% versus 66%, *p* = 0.120). Regarding the whole patient population, pre-transplant ECMO support was not a significant risk factor for 1-year mortality after adjusting for pre-transplant RRT (HR 1.355, 95% CI 0.822–2.233, *p* = 0.234). It was also not a significant risk factor for 1-year mortality in patients requiring RRT (Table 2).

### Long-Term Outcome

**FIGURE 2:**
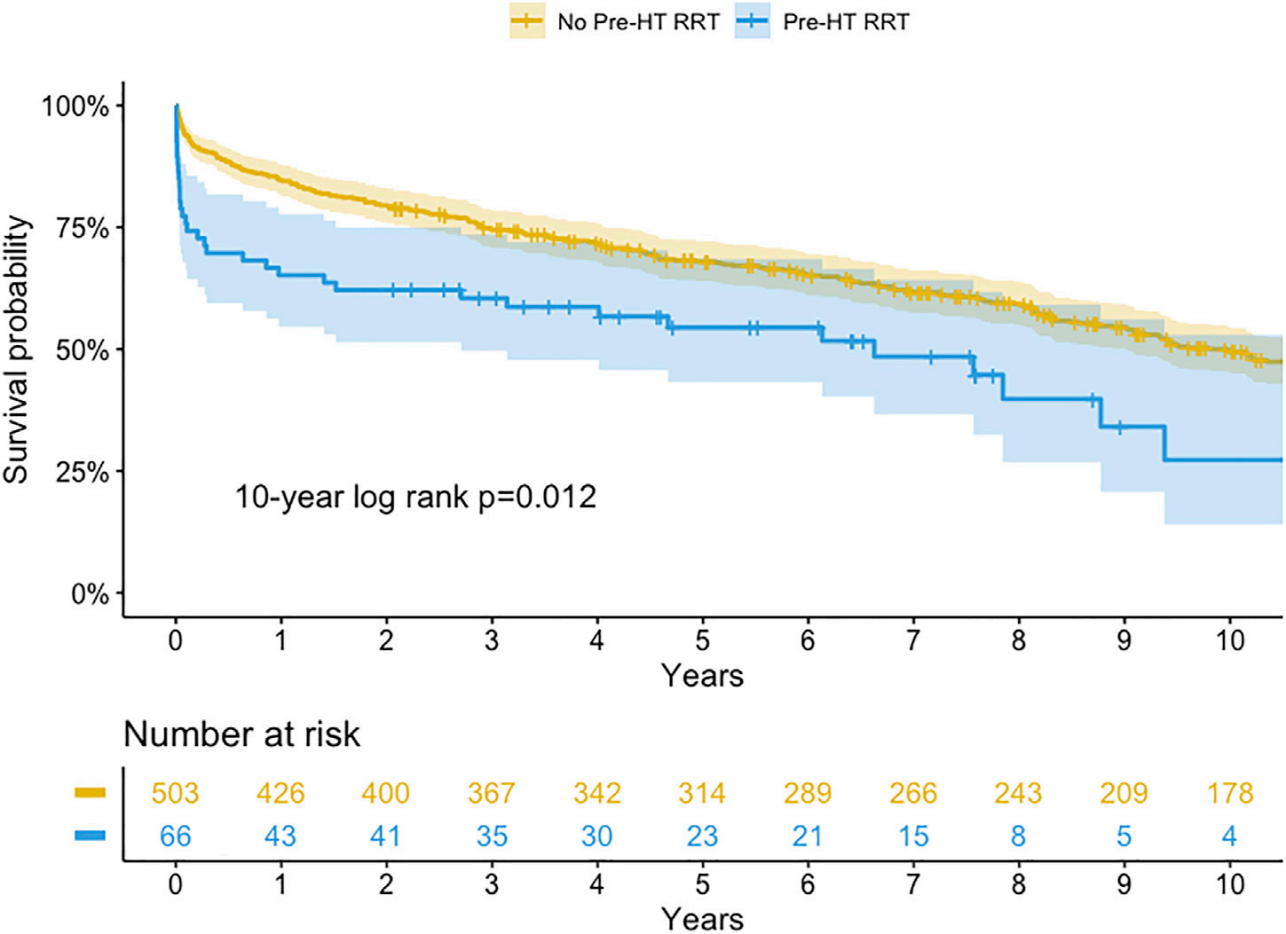
Kaplan-Meier survival curve for patients with and without renal replacement therapy (RRT) before heart transplantation (HT) (log-rank *p*-value = <0.001, 0.011, 0.012 at 1, 5, and 10-year, respectively).

**FIGURE 3:**
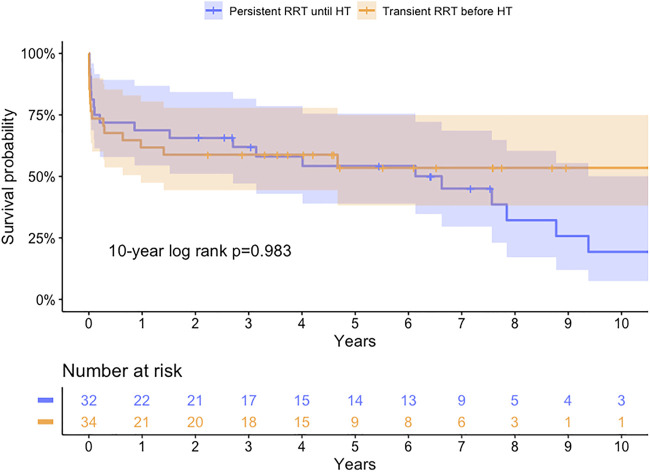
Kaplan-Meier survival curve for transient or persistent renal replacement therapy (RRT) before heart transplantation (HT) (log-rank *p*-value = 0.554, 0.558, 0.983 at 1, 5, and 10-year, respectively).

The long-term survival is shown in Figures 2 and 3. The 1-, 5-, and 10-year survival rates were 65.2, 54.4, and 27.3% in patients with RRT compared to 84.7%, 68.3%, and 51.1% in patients without RRT. Patients with RRT had a significantly lower overall survival rate than those without RRT (Log-rank *p*-value = 0.012 at 10-year).

We compared the RRT group to heart transplant patients on UNOS 1A status without RRT (*n* = 144). Survival was worse in patients with RRT than patients on UNOS 1A without RRT (65.2% vs 79.4%; 27.3% vs 44.7% at 1- and 10-year, Log-rank *p*-value < 0.001 and = 0.092, respectively).

For patients with RRT, the 1-, 5-, and 10-year survival rates were 61.8%, 53.5%, and 53.5% in the transient RRT subgroup compared with 68.8%, 54.2%, and 19.3% in the persistent RRT subgroup. The long-term survival rates were not different between subgroups (Log-rank *p*-value = 0.983 at 10-year).

**FIGURE 4:**
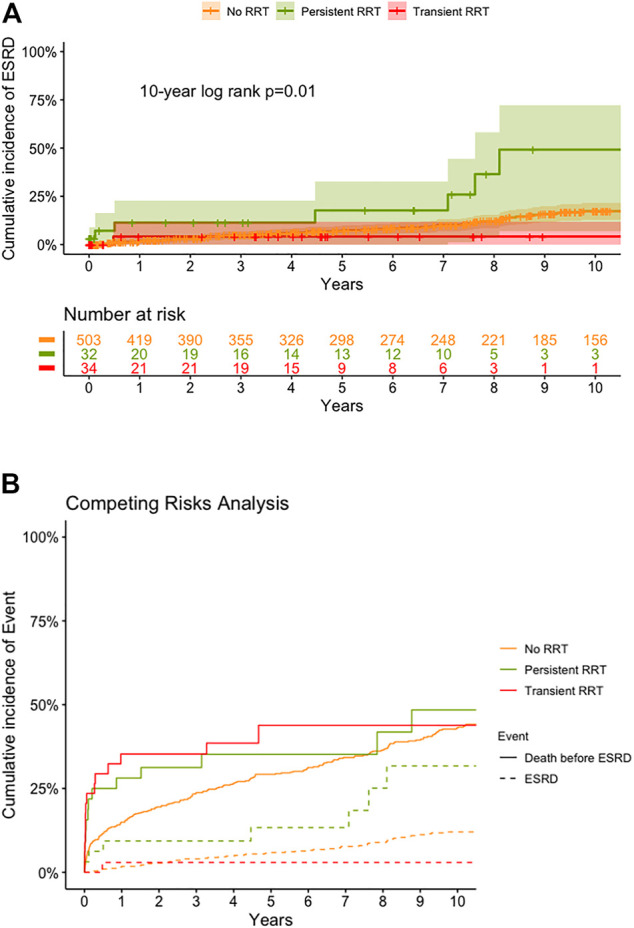
Cumulative incidence of end stage renal disease (ESRD) **(A)** and death before ESRD **(B)**, by competing risk analysis, by various groups. ESRD, end stage renal disease; RRT, renal replacement therapy; HT, heart transplantation.

The long-term renal outcome in each group is shown in Figure 4. The cumulative incidence of late ESRD in patients without RRT was 2.0, 7.4, and 17.3% at 1-, 5-, and 10-year (Figure 4A). For patients with RRT, the 10-year cumulative incidence of late ESRD in the transient RRT subgroup was 4.2% with only one patient developing ESRD at 6 months after HT. However, for patients with persistent RRT subgroup, the cumulative incidence of late ESRD was 11.4, 17.7, and 49.2% at 1-, 5-, and 10-year, which was much higher than that in patients without RRT and in the transient RRT subgroup. To correct the competing effect of death and ESRD, we analyzed the cumulative incidence of late ESRD and death before ESRD (Figure 4B). Patients with persistent RRT subgroup continued to have a much higher rate of late ESRD (*p* = 0.010).

Because the immunosuppressive regimen was changed during the observation period, we further stratified the patients into three groups according to different eras: 1989–2003 (*n* = 187), 2004–2009 (*n* = 173), and 2010–2018 (*n* = 209). Among patients from different eras, the long-term survival and the cumulative incidence of ESRD in a 10-year period showed no significant difference (log-rank *p* value = 0.6 and 0.059, respectively).

## Discussion

This is the first study to report on the clinical outcomes of isolated HT in patients who required RRT before HT. Previous studies have shown that pretransplant renal dysfunction was associated with a high incidence of postoperative ESRD and RRT after HT (15). Pretransplant RRT was also associated with a poor outcome after HT (5, 6, 15). However, patients with cardiogenic shock complicating AKI could have renal function recovery following hemodynamic stabilization (16), and early RRT could improve survival and gain a better recovery of renal function after HT (17). The recovery of renal function depends on several factors including patient comorbidity and MCS device-related infection, hemolysis, and thromboembolic events (16, 18). For transplant surgeons, it was very difficult to predict the renal outcome after HT and allocate organ replacement to those transplant candidates with complicating AKI and requiring RRT before HT.

In this study, patients requiring RRT before HT had poor short-term and long-term survival. Several recipient variables have been recognized as risk factors for mortality after HT, including old age, resternotomy, hospitalization, intubation, low estimated glomerular filtration rate, serum total-bilirubin level >2 mg/dl, and use of MCS (5,19). In this study, more than 80% of patients requiring RRT had a high rate of UNOS 1A status, resternotomy surgery, and MCS use. All these factors could contribute to the inferior survival observed in patients requiring RRT.

The clinical outcomes following HT have improved over time (20). The 10-year survival rate among all HT patients in our hospital was >50%. However, in patients with pre-transplant RRT, the 10-year survival rate was comparatively low. As shown in Figure 2, most of the mortality in patients requiring RRT occurred within 1 year after HT. After 1 year, the rate of survival decline was not different between patients with and without RRT. Therefore, it was imperative to identify the risk factors associated with 1-year mortality after HT in patients with pre-transplant RRT. Careful patient selection could achieve better survival after HT in this critical situation. In this study, we identified pre-transplant hyperbilirubinemia, post-transplant RRT, and post-transplant early BSI as the independent risk factors of 1-year mortality. Hyperbilirubinemia was the most significant pretransplant predictor of 1-year mortality after HT.

The occurrence of liver dysfunction was not rare in patients with heart failure and probably even more common in heart transplant candidates. Ischemic liver hypoperfusion and hepatic congestion were the two major pathogenic mechanisms in cardiogenic shock and congestive heart failure (21). Heart failure complicating with liver dysfunction adversely affected prognosis. Furthermore, preoperative liver dysfunction had a significant impact on the survival of patients after HT (22). The presence of pre-transplant hyperbilirubinemia indicated an advanced heart failure and a combination of renal failure and liver dysfunction implied an even worse outcome after HT (23).

According to the ISHLT report, acute graft failure was the most common cause of mortality within the first 30-days after HT (24). In this study, the major cause of 30-day mortality was an infection in patients with pre-transplant RRT. Previous studies have reported that pre-transplant RRT was a major risk factor of post-transplant BSI in HT (2, 25). Both use of RRT and MCS before HT would further increase the risk of BSI before and after HT (12, 26). In our study, 17 (26%) patients with pre-transplant RRT had pre-transplant BSI, and 8 of them (12%) had a positive blood culture within 2-week before HT. Early BSI after HT was one of the major risk factors of 1-year mortality.

CKD and dialysis-dependent ESRD were major long-term complications after HT. The 10-year incidence of developing ESRD was 6% in ISHLT reports (22). We have previously reported that Chinese heart recipients had a higher incidence of developing CKD and dialysis-dependent ESRD than recipients from other countries. The cumulative incidence of late dialysis-dependent ESRD was 16% at 10-year after HT, and the prognosis was poor after RRT (27, 28). In this study, among patients with persistent RRT before HT, 72% of them had renal function recovery after HT and were discharged without RRT. However, the cumulative incidence of developing dialysis-dependent ESRD was 49% at 10-year. Whether a combined HKT could improve survival in these cases was unknown. However, donor shortage made combined organ transplantation difficult. The clinical outcomes of combined HKT were unsatisfactory in heart transplant candidates in UNOS status IA and requiring resternotomy surgery (8, 13). Considering the potential of renal recovery after HT and donor shortage, a staged approach with renal transplant after HT was advisable for those heart transplant candidates requiring RRT after isolated HT (29).

This study has several limitations. First, it was retrospective and the details of residual renal function could not be obtained completely. Second, the small case numbers limited the statistical power to have more independent risk factors of 1-year mortality. Third, the study spanned almost 3 decades, which introduced limitations since there have been significant inevitable practice changes in the management of cardiogenic shock, AKI, and HT over time. Fourth, based on our data, the high incidence of ECMO support among patients requiring RRT may influence renal recovery and outcome. However, the small case number and heterogeneous type of MCS did not allow for further exploration of the impact of ECMO. As there is a possibility of bias, a large registry, propensity score-matched, and multi-center studies are warranted for further exploration of these issues.

This was the first study to focus on the long-term outcomes for patients requiring RRT before HT. Careful patient selection and proper postoperative management in this critical situation are important to achieving better survival rates after HT. Heart transplant candidates with pretransplant RRT and hyperbilirubinemia should be carefully re-evaluated for the eligibility of HT because of an inferior survival rate. Prevention and management of BSI after HT were crucial in patients requiring RRT before HT.

### Conclusion

For isolated HT, patients with RRT before HT had a worse short-term and long-term survival. Renal function recovered after HT in the majority of operative survivors. Patients with persistent or transient RRT before HT had similar long-term survival. However, patients with persistent RRT until HT had a higher incidence of late ESRD requiring RRT.

## Data Availability

The data that support the findings of this study are available on request from the corresponding author.

## Abbreviations

AKI, acute kidney injury; ECMO, extracorporeal membrane oxygenation; ESRD, end stage renal disease; HT, heart transplantation; MCS, mechanical circulatory support; RRT, renal replacement therapy; UNOS, united network for organ stage; VAD, ventricular assisted device.
